# Development of an Animal Model for Traumatic Brain Injury Augmentation of Heterotopic Ossification in Response to Local Injury

**DOI:** 10.3390/biomedicines11030943

**Published:** 2023-03-18

**Authors:** Chandrasekhar Kesavan, Gustavo A. Gomez, Sheila Pourteymoor, Subburaman Mohan

**Affiliations:** 1 Musculoskeletal Disease Center, VA Loma Linda Healthcare System, Loma Linda, CA 92357, USA; 2 Department of Medicine, Loma Linda University, Loma Linda, CA 92354, USA; 3 Orthopedic Surgery, Loma Linda University, Loma Linda, CA 92354, USA

**Keywords:** repetitive mild TBI, mice, tendon injury, hypoxia, heterotopic ossfication, pericytes

## Abstract

Heterotopic ossification (HO) is the abnormal growth of bone in soft connective tissues that occurs as a frequent complication in individuals with traumatic brain injury (TBI) and in rare genetic disorders. Therefore, understanding the mechanisms behind ectopic bone formation in response to TBI is likely to have a significant impact on identification of novel therapeutic targets for HO treatment. In this study, we induced repetitive mild TBI (mTBI) using a weight drop model in mice and then stimulated HO formation via a local injury to the Achilles tendon or fibula. The amount of ectopic bone, as evaluated by micro-CT analyses, was increased by four-fold in the injured leg of mTBI mice compared to control mice. However, there was no evidence of HO formation in the uninjured leg of mTBI mice. Since tissue injury leads to the activation of hypoxia signaling, which is known to promote endochondral ossification, we evaluated the effect of IOX2, a chemical inhibitor of PHD2 and a known inducer of hypoxia signaling on HO development in response to fibular injury. IOX2 treatment increased HO volume by five-fold compared to vehicle. Since pericytes located in the endothelium of microvascular capillaries are known to function as multipotent tissue-resident progenitors, we determined if activation of hypoxia signaling promotes pericyte recruitment at the injury site. We found that markers of pericytes, NG2 and PDGFRβ, were abundantly expressed at the site of injury in IOX2 treated mice. Treatment of pericytes with IOX2 for 72 h stimulated expression of targets of hypoxia signaling (*Vegf* and *Epo*), as well as markers of chondrocyte differentiation (*Col2α1* and *Col10α1*). Furthermore, serum collected from TBI mice was more effective in promoting the proliferation and differentiation of pericytes than control mouse serum. In conclusion, our data show that the hypoxic state at the injury site in soft tissues of TBI mice provides an environment leading to increased accumulation and activation of pericytes to form endochondral bone.

## 1. Introduction

Traumatic brain injury (TBI) is becoming a major cause of death, disability, and morbidity worldwide and a significant public health concern. Each year, 1.4 million cases of brain injury or TBI are reported in the United States (CDC.gov/Traumatic brain injury) [1,2]. TBI affects all age groups and occurs after accidents, sports injuries, military conflicts, and after falls. Many individuals diagnosed with TBI are left with short or long-term neurological problems, have experienced hospitalization, require special care, and live with a poor quality of life.

TBI can be classified based on the severity as mild, moderate, or severe depending on the location of the injury and on the time of lost consciousness [3,4,5]. Mild TBI is characterized by loss of consciousness for a few minutes and may or may not cause a confused or disoriented state. In moderate TBI, patients experience a loss of consciousness that lasts from several hours to weeks. Patients who experience moderate TBI may show signs of physical, cognitive, or behavioral changes that may last months or can be permanent. In severe TBI, there is a physical injury to the skull and brain that is life-threatening and can cause patients to experience memory loss, speech problems, sensory impairments, psychological disorders, etc. [3,6].

Individuals who are diagnosed with TBI experience different types of short- and long-term comorbidities, such as post-traumatic stress disorder (PTSD), cognitive disorders, back pain, depression, hypertension, anxiety, skeletal fractures, sleep disorders, panic attacks, osteoarthritis, and diabetes [7,8,9,10,11,12,13,14,15]. Importantly, TBI patients with musculoskeletal injuries (fracture, leg amputation) tend to develop bone (heterotopic ossification [HO]) in soft tissues at or near the injury site [16,17]. HO is very common in muscles surrounding the hip, joints and in the knee. Furthermore, the rate of HO in these muscles is substantially higher after combat in military personnel than in the civilian population [18]. The formation of HO in the context of an associated head injury has been reported in 20% of patients with forearm fractures, in 52% of patients with femoral shaft fractures, and in 15–30% of patients with spinal cord injuries [19].

Besides HO that is acquired in the setting of physical trauma, formation of ectopic bone is a common occurrence in fibrodysplasia ossificans progressive (FOP) that is primarily genetically driven. Genetic mutations in the gene that codes for activin A receptor type I (ACVR1) have been shown to contribute to the FOP disorder [20,21]. There has been a great deal of controversy regarding the exact nature of progenitors for osteoblasts involved in FOP and HO formation. Different sources of osteogenic precursors including mesenchymal stem cells in the bone marrow, pericytes, satellite cells, interstitial cells between muscle fibers, circulating stem cells and nerve stem cells have been proposed [22]. A common niche for mesenchymal progenitors is the perivascular space, and there is considerable evidence in the literature for regenerative potential of pericytes when applied to bone. Pericytes, characterized by high expression of neural/glial antigen 2 (NG2) and platelet-derived growth factor rector β (PDGFRβ), have been implicated in the repair and regeneration of tissues damaged by injury [23].

Our understanding of how HO is formed at the injury site in TBI patients is rather limited. In this regard, studies have shown that the brain releases several osteogenic and inflammatory factors including bone morphogenetic proteins (BMPs), fibroblast growth factors (FGFs), interleukin (IL)6, calcitonin gene-related peptide (CGRP), oncostatin M, and substance P [24] in response to injury that could exert a direct or indirect role in differentiating local stem cells into osteogenic lineage cells, resulting in the development of mineralized HO formation in soft tissues. Presently, surgery, radiation therapy, anti-inflammatory drugs, agonist or inhibitors against target receptors, and bisphosphonate treatment are used in the clinic to reduce the HO formation. However, additional surgery can result in bleeding and infection and radiation increases the risk of malignancy. Anti-inflammatory drugs cause GI problems and long-term use of bisphosphonate results in skeletal fragility and fracture risk. Furthermore, these therapies are limited in effectiveness and the reoccurrence of HO is high. Towards the goal of understanding the pathophysiology of TBI-induced HO, which is essential for the future development of HO therapies, we focused our efforts to develop an animal model to study TBI augmentation of HO formation in response to local bone injury.

## 2. Materials and Methods

### 2.1. Mild Traumatic Brain Injury (mTBI)

The nine-week-old male C57BL/6J mice were purchased from Jackson Laboratory and randomized based on their body weight to minimize bias and housed for one week for acclimatization. At 10 weeks, mTBI was induced as previously reported [25]. A 75 g brass weight was used to generate a consistent impact on the skull. Under isoflurane anesthesia, mice were subjected to repeated mTBI from a height of 1.5 m, once per day, for four consecutive days. To obtain a consistent impact on the mouse skull, a tube was positioned above the mouse calvaria, between the ears. Control mice received isoflurane anesthesia only. All mice survived for the duration of the experiment, and no paralysis or skull fractures were observed. All procedures were approved by the Institutional Animal Care and Use Committee of the VA Loma Linda Healthcare System, Loma Linda, CA, USA (Permit #0046/1119).

### 2.2. HO Model

Achilles tenotomy: Two weeks after impact (i.e., at 12 weeks), the mice were given a subcutaneous injection of buprenorphine (VA Loma Linda Healthcare System Pharmacy) one time prior to surgery (from 0.05 to 0.1 mg/kg body weight). We chose buprenorphine because it is widely prescribed in Veterinary medicine to help reduce pain in animals and, furthermore, it is fast acting and very effective. Twenty minutes after buprenorphine administration, the mice were anesthetized with isoflurane using a nose cone. Mice were placed on a heating pad platform in a lateral position. A small longitudinal incision was performed on the skin at the medial gastrocnemius muscle site to expose the right tibia. The micro-scissors were inserted into the medial gastrocnemius muscle site like a needle and muscle was damaged. Simultaneously, the Achilles tendon was dissected at the distal region of right tibia [26]. The skin was subsequently closed using a non-absorbable suture. The mice were assessed twice daily for signs of unrelieved pain.

Fibular injury: In this method, mice were prepared as mentioned above for an Achilles tenotomy. A small longitudinal incision was performed in the skin at the medial gastrocnemius muscle site in the right tibia. A micro-scissors was inserted into the medial gastrocnemius muscle site like a needle and the fibula was cut. The skin was closed with a non-absorbable suture and the cut was validated by radiography using the Ultra Focus Faxitron (Faxitron Bioptics LLC., Tucson, AZ, USA).

### 2.3. Dual X-ray Absroptiometry

The mice were anesthetized using 2–4% isoflurane, 0.5 L/minute oxygen inhalation. During this time, the mice were positioned in the DEXA chamber and X-ray was performed to access if mice developed HO in the injured leg compared to the non-injured leg using the Ultra focus Faxitron (Hologic, Tucson, AZ, USA) [27].

### 2.4. Micro Computed Tomography (µ-CT)

For *ex-vivo* µ-CT evaluations, a high-resolution tomography image system (viva CT40; Scano Medical AG, Bruttisellen, Switzerland) was used to measure ectopic bone formation at the injured gastrocnemius muscle site as previously described in [28]. Routine calibration was performed once per week using a three-point calibration phantom corresponding to a density range from that of air to cortical bone. The legs with intact bone and muscle were scanned at a resolution of 10 µm with a 55 kVp X-ray. After acquiring the radiographic data, images were reconstructed using the 2-D image software provided by Scanco. Every 10 sections of the ectopic bone and trabecular bone were outlined, and the intermediate sections were interpolated with the contouring algorithm to create a volume of interest, followed by the three-dimensional analysis. Parameters such as total volume (TV, mm^3^), bone volume (BV, mm^3^), bone volume fraction (BV/TV, %), apparent density (mg HA/cm), trabecular number (Tb. N, mm^−1^), trabecular thickness (Tb. Th, mm), and trabecular space (Tb. Sp, mm) were evaluated in the impacted and non-impacted groups.

### 2.5. Histology

The right and left leg with intact tibia and muscles were isolated from the euthanized mTBI mice and fixed in 10% neutral buffered formalin for 48 h. The legs were rinsed with 1X phosphate buffered saline (PBS) to remove the formalin, were subsequently decalcified using 14% ethylenediaminetetraacetic acid (EDTA) and embedded for paraffin sectioning. Thin six micron cross-sectional sections were cut and stained for ectopic bone with Goldner’s trichrome stain and were subsequently examined under an Olympus BH-2-fluorescence/bright field microscope [28]. Subsequently, the sections were processed for immunohistochemistry to measure the expression levels of bone marker genes in the injured vs. non-injured leg.

### 2.6. HO Induction and IOX2 Treatment

Fourteen-week-old male C57BL/6J mice were subjected to a fibula-muscle injury as described above in the methods. The second day after injury, these mice were given intraperitoneal injections of IOX2 (Bio-Techne Corporation, Minneapolis, MN, USA), 17.5 mg/kg daily (5 days/week) for a period of 8 weeks. Twenty-two-week-old mice were euthanized, and tissues were collected for quantitating HO and skeletal parameters by micro-CT and histology.

### 2.7. Pericytes Cultures

Pericytes were isolated as described in [29]. Three-week-old mice were euthanized, and brain tissue was collected and washed in Alpha Minimum Essential Medium (αMEM). The brain tissue was minced and washed by centrifugation at 1200 rpm for 5 min. The minced brain tissue was incubated in an enzymatic solution—30 U/mL papain, 40 ug/mL DNase I in Earl’s Balanced Salt Solution (EBSS)—for 70 min at 37 °C. The homogenized solution was passed through an 18-gauge needle and subsequently through a 21-gauge needle for 10 times. The homogenized brain cells were mixed with 1.7 volumes of 22% bovine serum albumin (BSA) in 1X PBS and centrifuged at 4000 rpm for 10 min. The supernatant layer containing the cells was carefully removed, and the cell pellet was re-suspended in 5 mL of endothelial cell growth medium (ECGM) consisting of Ham F12 supplemented with 10% fetal bovine serum (FBS), heparin, ascorbic acid, L-glutamine, and penicillin/streptomycin and centrifuged for 5 min at 1200 rpm. The cells were re-suspended in ECGM and plated on 6-well plates that were pre-coated with type-I collagen for 20 h at 37 °C. After 7–9 days, or once the cells reached confluency, they were passed 1:4 onto a fresh collagen-coated 6-well plate. Cells from the first two passages were maintained in ECGM, while the third passage cells were maintained in pericyte medium (ScienCell Research Laboratories, Carlsbad, CA, USA) containing 2% FBS.

### 2.8. mTBI Serum and Osteogenic Activity

Calvaria were isolated from newborn C57BL/6J mice and digested with type-I collagenase twice for 2 h. The cells were collected by centrifugation and seeded in culture plates for 48 h in αMEM containing 10% calf serum and penicillin (100 U/mL). Once the cells were 80% confluent, they were passed and plated (5000 cells/well) for proliferation and alkaline phosphatase activity (ALP) assay. For proliferation assays, the cells were cultured in serum containing medium for 24 h, followed by serum free medium in αMEM containing 0.1% BSA for 24 h. After this incubation period, the cells were cultured in αMEM with 0.1% BSA for 48 h in the presence or absence of varying doses of serum derived from mTBI mice or non-impacted control mice. We treated the control group with 1X PBS (vehicle). Forty-eight hours later, the cells were subjected to a proliferation assay using a Cy-Quant dye Kit (Life Technologies, Carlsbad, CA, USA).

For ALP assessment, pericytes or ST2 stromal cells and MC3T3-E1 were cultured in medium with serum for 24 h followed by serum free media for 24 h. Subsequently, cells were incubated for 72 h in αMEM containing 0.1% BSA, ascorbic acid (100 µg/mL), and β-glycerophosphate (10 mM) in the presence or absence of mTBI serum, control serum, or vehicle. The cultures were terminated 72 h after treatment and ALP activity was assessed by measurement of para-nitrophenol (Sigma-Aldrich, St. Louis, MO, USA) measurement on a plate reader.

The student t-test was used to compare if number of HO measured by micro-CT were significantly different between the groups. We also used a student t-test to determine if trabecular bone parameters at the secondary spongiosa and if the HO bone density was significantly different between control and mTBI mice. A *p*-value of <0.05 was considered statistically significant. The data were presented as the mean ± standard error of mean (SEM).

## 3. Results and Discussion

**Figure 1:**
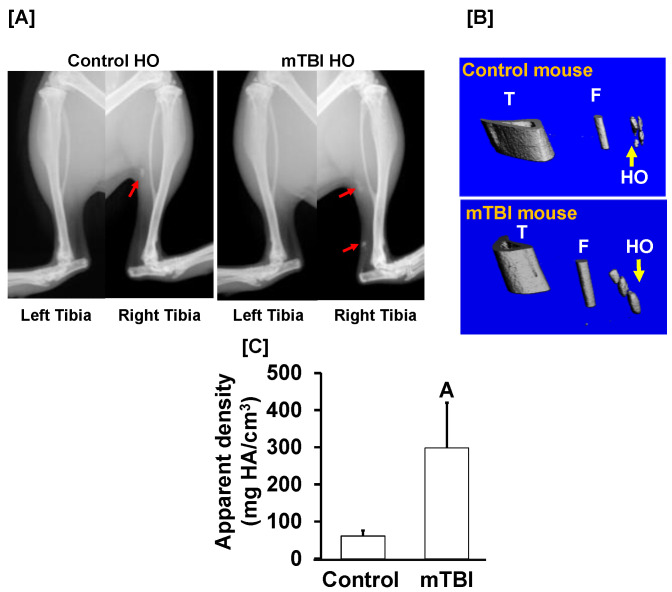
X-ray image and quantitative data describing HO. (**A**) The X-ray image shows HO in the control and mTBI mice 8 weeks after injury. The image was obtained at 40 µm resolution. The red arrow corresponds to HO, (**B**) Micro-CT image of HO in the control and mTBI mice, and (**C**) quantitation of apparent density (BMD) of HO in the control and mTBI mice. Values are the mean ± SEM. N = 5, ^A^ *p* = 0.08 vs. control.

Recent studies have found that the incidence of HO in patients with TBI is about 20%, but the rate exceeds 50% when TBI is concomitant with femur fracture [17,19]. To determine if the association between TBI and fracture HO incidence can be reproduced in a mouse model of mild TBI, we subjected mice to a weight drop model to induce mild TBI, followed by a local Achilles tenotomy injury to induce HO, which was evaluated at different times. We found, by DEXA analysis, that the mTBI mice subjected to Achilles tenotomy showed a greater amount of ectopic bone compared to non-impacted-injured mice (control mice) at 8 weeks after injury (Figure 1A). However, as expected, there was no evidence of ectopic bone in the injured leg of either mTBI or control mice. Micro-CT analysis of HO revealed a four-fold increase in the amount of ectopic bone (BMD) in the mTBI mice compared to control mice 8 weeks after injury (Figure 1B,C). While we did not see differences in the number of HO sites (1.8 vs. 1.7 average) between the mTBI and control groups, our findings demonstrate that the amount of ectopic bone formed is increased in mTBI mice in response to a local tissue injury.

**Figure 2:**
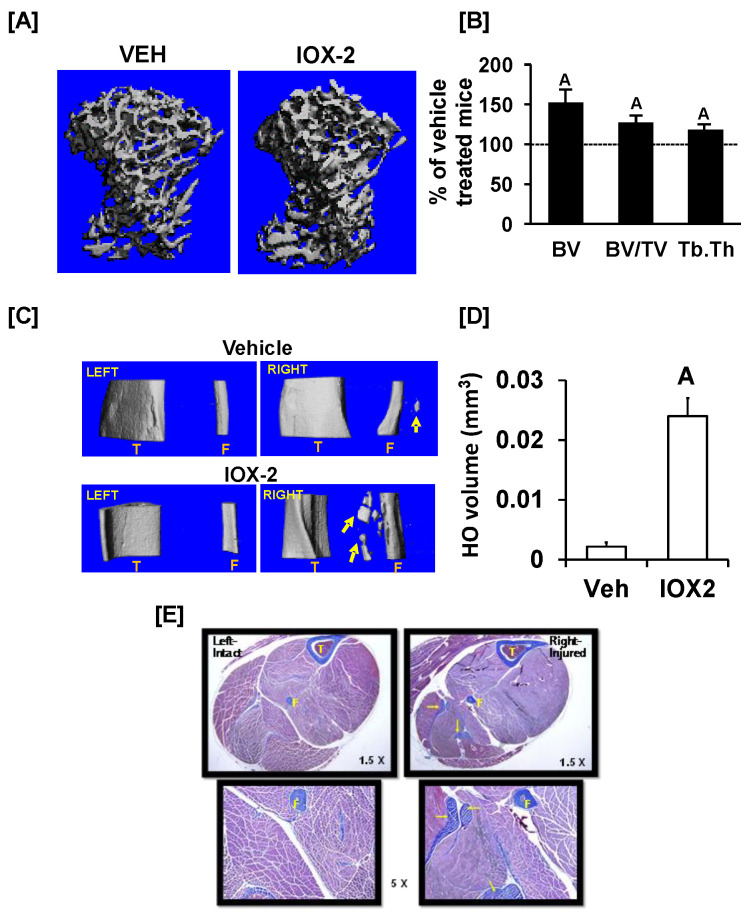
Illustration and quantitation of trabecular bone parameters and HO formation in the fibula-muscle injury mice response to IOX2 treatment. (**A**) Micro-CT image of trabecular bone mass at the secondary spongiosa in the fibula-muscle injury mice treated with vehicle or IOX2, (**B**) quantitation of trabecular bone parameters in 22-week-old fibula-muscle injury mice treated with vehicle or IOX2, (**C**) micro-CT image of HO indicated with arrows near the fibula cut region, (**D**) quantitation of HO volume by micro-CT, and (**E**) trichrome staining of HO near the fibula cut region by histology. T—tibia and F—fibula. Values are the mean ± SEM. N = 5, ^A^ *p* < 0.05 vs. control.

The occurrence of HO after injury involves surrounding tissues creating an environment favorable for cellular processes to take place, release of systemic factors at the site of injury and trans-differentiation of non-bone cells into osteogenic lineage cells. These combined processes are mediated by multiple molecular and cellular events which remain largely unclear. Other studies have identified BMP and mammalian target of rapamycin (mTOR) signaling pathways playing a significant role in the pathogenesis of HO [30,31,32,33,34]. In addition to these signaling pathways, there are indisputable evidence that the process of HO is highly dependent on angiogenesis that also plays an important role in the process of endochondral ossification [35,36]. Angiogenesis is mediated by multiple signaling pathways, among which hypoxia-inducible factor-1 alpha (HIF1α) has gained significance because (1) HIF1α signaling is activated during hypoxia (a condition that occurs in the soft tissue after injury) [37]; (2) HIF1α promotes transcription of key genes such as vascular endothelial growth factor (*Vegf)* and erythropoietin (*Epo*) which are involved in stimulating angiogenesis [38]; and (3) Inhibition of HIF1α has been shown to prevent trauma-induced HO [39]. The stability and function of HIF is regulated post-translationally by hydroxylation of specific prolines in the presence of oxygen by prolyl hydroxylase domain (PHD) enzymes (PHD1, PHD2, PHD3), which are the primary cellular oxygen censors. PHD2 has been shown to be the key isoform responsible for HIF1α regulation in many cell types, including bone cells. Our group has reported that disruption of the *Phd2* gene in chondrocytes encouraged chondrocyte differentiation and osteoblast function and this effect was, in part, mediated via upregulation of HIF1α signaling [40,41,42]. Based on these and other data, we predicted a key role for hypoxia signaling in promoting HO formation. To determine the role of HIF1α signaling in the development of HO, we subjected the mice to a fibular injury to induce HO, and then treated these mice with IOX2 (a chemical inhibitor of PHD2) or vehicle for a period of 8 weeks (5 days/week). We chose a fibula injury model to induce HO because it is feasible to do with relative ease in rodents and this model also mimics HO formation in humans [43]. Micro-CT analysis showed an increase in trabecular bone mass at the metaphysis in mice treated with IOX2 (Figure 2A). Quantitative analysis revealed a 20% increase in BV and BV/TV in mice treated with IOX2 that was caused by an increase in trabecular thickness (Figure 2B), a measure of osteoblast differentiation. Figure 2C shows representative micro-CT images revealing an increased number of HO sites (Figure 2C). The amount of ectopic bone, as revealed by HO volume, was increased by nearly 10-fold at the fibula injury site in the mice treated with IOX2 compared to mice treated with vehicle (Figure 2D). Consistent with this data, histological sections stained with trichrome revealed evidence for a greater amount of blue stained ectopic bone in response to injury in IOX2 treated control mice compared to vehicle treatment (Figure 2E). Together, these data demonstrate that HIF1α signaling, a major stimulator of angiogenesis, is an important regulator of HO in response to injury.

**Figure 3:**
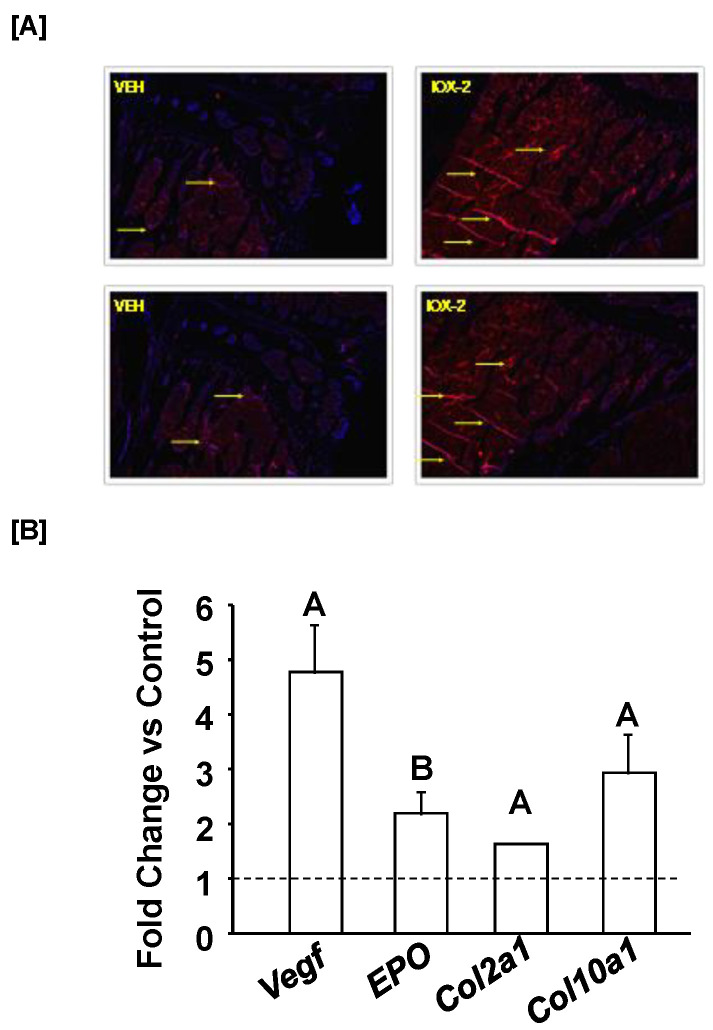
Expression patterns of pericytes and bone markers in response to IOX2 treatment. (**A**) Expression of pericyte markers by immunohistochemistry at the injured site of control mice treated with IOX2 or vehicle and (**B**) Three days treatment of IOX2 increased expression of angiogenesis and endochondral bone markers in pericytes. Values are mean ± SEM. Arrows show expression (red) of NG2 and PDGFRβ. ^A^ *p* < 0.05; ^B^ *p* = 0.08 vs. vehicle control.

In terms of cell populations contributing to HO, studies using inherited conditions such as FOP have identified various candidate precursor cell populations, including endothelial precursors, circulating osteogenic precursors, satellite cells, mesenchymal stem cells, and pericytes. Our focus in this study on pericytes was based on their identification as multipotent-resident progenitors and their anatomical location encircling the endothelium of microvascular capillaries. If pericytes are an important source of bone cells, we anticipate a greater number of pericytes at the site of injury in mice treated with IOX2. To test this, we performed IHC at the fibula-gastrocnemius site of muscle injury in the control mice treated with IOX2 for a period 8 weeks. Since IOX2 inhibits PHD2 and, thereby increases HIF1α levels, we expected to observe increased vascularization. Accordingly, by IHC, we found increased expression of NG2 and PDGFRβ, well-established markers of pericytes, at the injury site of IOX2 treated mice compared to vehicle treated control mice (Figure 3A). While our findings show the presence of pericytes at the site of injury, it is not clear if pericytes are differentiating into osteogenic lineage cells and, thereby inducing HO. We, therefore, isolated pericytes from mice brain and treated the cells with IOX2 for 72 h *in vitro*. We used 3 week-old male and female mice for isolation of adequate number of pericytes for our in vitro experiments since the replication potential of pericytes from prepubertal young mice is much higher compared to pericytes from 3-month-old adult mice. At three weeks of prepubertal age, no gender differences were seen in mice. Interestingly, we found IOX2 treatment increased expression of angiogenesis markers (*Vegf*, *Epo*) and expression of chondrocyte marker genes (collagen (*Col) 2*, *Col10*), providing evidence that pericytes possess the ability to differentiate into osteogenic lineage cells and, thereby contributing to HO formation (Figure 3B).

**Figure 4:**
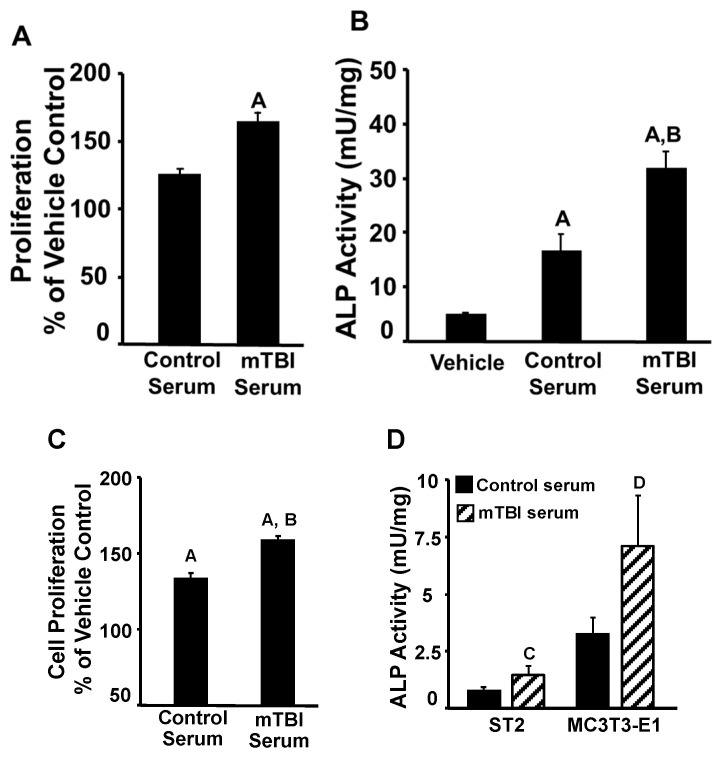
Effect of control and mTBI serum on cells. Effect of mTBI or control serum on proliferation in pericytes (**A**) and alkaline phosphatase (ALP) activity in pericytes (**B**), primary osteoblasts (**C**) and ST2 stromal cells (**D**). Values are mean ± SEM, ^A^ *p* <0.05 vs. vehicle control, ^B^ *p* < 0.05, ^C^ *p* = 0.07, ^D^ *p* = 0.1 vs. control serum.

Recent studies have shown that TBI causes blood-brain barrier dysfunction, resulting in the release of various factors (cytokines and growth factors) systemically [44,45,46]. Since pericytes express a broad range of receptors, we predict some of these factors released into blood can play an important role in differentiating pericytes into osteogenic cells. To test this prediction, we treated pericytes with 1% serum from mTBI and control mice and found that both proliferation (Figure 4A) and differentiation (reflected by ALP activity) (Figure 4A,B) were increased by serum collected from TBI mice compared to control mice. Similarly, serum collected from TBI mice increased proliferation in primary osteoblasts (Figure 4C) and was more effective in inducing ALP activity in ST2 stromal cells and MC3T3-E1 pre-osteoblasts (Figure 4 D). Thus, the findings of this study provide a potential mechanism that hypoxia signaling and factors released into the serum, as a consequence of mTBI, are involved in the development of HO at the injury site by acting on stem cells (e.g., progenitors such as pericytes) to promote their proliferation and differentiation into osteogenic lineage cells.

## 4. Conclusions

In conclusion, our data show that the hypoxic state at the injury site in soft tissues of TBI mice provide an environment leading to increased accumulation and activation of pericytes to form endochondral bone.

## Data Availability

Raw data are available upon request.
